# Male African elephants discriminate and prefer vocalizations of unfamiliar females

**DOI:** 10.1038/srep46414

**Published:** 2017-04-19

**Authors:** Angela S. Stoeger, Anton Baotic

**Affiliations:** 1 Mammal Communication Lab, Department of Cognitive Biology, Althanstrasse 14, 1090, University of Vienna, Austria

## Abstract

Gaining information about conspecifics via long-distance vocalizations is crucial for social and spatially flexible species such as the African elephant (*Loxodonta africana*). Female elephants are known to discriminate individuals and kin based on acoustic cues. Specifically, females approached the loudspeaker exclusively with playbacks of familiar individuals with high association indexes, intentionally fusing with their affiliates. For males, which are less bonded, gathering social information via vocalizations could still have important implications, but little is known about their vocal discrimination skills. We experimentally tested the ability of male African elephants to discriminate the social rumbles of familiar (from the same population) versus unfamiliar females. Male elephants discriminated and preferentially moved towards the rumbles of unfamiliar females, showing longer attentive reactions and significantly more orientating (facing and approaching the speaker) behavior. The increased orientating response of males towards playbacks of unfamiliar females is converse to the reaction of female subjects. Our results provide evidence that male elephants extract social information from vocalizations, yet with a different intention than females. Accordingly, males might use social cues in vocalizations to assess mating opportunities, which may involve selection to identify individuals or kin in order to avoid inbreeding.

Acoustic communication in socially and spatially flexible species occurs in networks in which multiple signalers and receivers are within signaling range^1^. In such species, long-distance vocal signals are often an important means to maintain connectivity and to gain information about conspecifics^2^,^3^,^4^. The acoustic information available in a signal needs to be decoded and processed by receivers, which, in some species, is a complex cognitive task^5^. The ability to discriminate between vocalizations from different individuals has been documented in several mammalian species^6^,^7^,^8^ and is suggested to impart functional advantages. Discriminating kin or socially affiliated individuals can be crucial for survival, and assessing the quality or eligibility of potential mating partner can be critical for reproductive success^1^.

The African elephant (*Loxodonta africana*) exhibits a fission fusion society^9^ and is one example of a social, yet spatial dispersed species. The ranging pattern of males and females can cover huge areas (in Kenya averaging 225 km^2^ (14–783 km^2^)^10^ depending on habitat type and size as well as seasonal effects, and hundreds of individuals can be encountered opportunistically^11^. Females are the more social sex, maintaining close bonds between the members of a family unit (composed of adult females and their offspring), and are further linked to other families, forming bond groups^9^,^12^. Males leave their natal family at an average of 14 years^11^, but also form all-male alliances or associations, although the bonds are less tight than those of female family members^13^,^14^,^15^.

Elephants produce low-frequency (fundamental frequencies ranging from 10 to 35 Hz), harmonically rich vocalizations termed rumbles used in short- and long-distance communication^16^,^17^,^18^,^19^. These rumbles transmit information about identity, reproductive state, arousal, age and size of the caller^16^,^19^,^20^,^21^,^22^,^23^. Female elephants use such rumbles to identify and maintain contact with socially affiliated individuals^20^,^24^. Playback experiments in the Amboseli National Park revealed that female elephants could distinguish the rumbles of family and bond group members from those outside these categories^20^. More specifically, approaches of females to the loudspeaker were exclusively associated with playbacks of familiar individuals with high association indexes (i.e. family or bond group members), most likely with the intention of fusing with their social affiliates^20^. Those authors further estimate that subjects are familiar with the vocalizations of a mean of 14 families in the population, containing around 100 adult females in total^20^. Social discrimination in females seems possible over distances of 2.5 km, but was usually achieved reliably over distances of 1–1.5 km^16^.

While some information is available on the acoustic structure and the information content of male elephant rumbles^21^,^25^,^26^, little is known about male vocal perception and discrimination abilities. Similar to other mammals^27^, however, male elephants are capable of perceiving female reproductive state from vocal cues^28^. The differences in sociality of male and female elephants^9^ raise the question whether males are as skilled in extracting social information from vocalizations as their female counterparts. This is based on the consideration that cognitive abilities seem to be adapted to the specific needs of the organism, often resulting in differences between the sexes of a species^29^,^30^,^31^.

Vocally distinguishing and assessing individuals from a distance could be relevant for male elephants for several reasons, including competitor and mate assessment. Sex-biased dispersal in elephants does not lead to the complete separation of male and female relatives^11^,^32^. Accordingly, even when males are fully independent and mature, kin appear to be commonly available as potential mates in natural populations; sexually active males return to core female areas, including their own natal area, to find mates^32^. Competition among males for mates is intense and the costs of inbreeding may be higher for males than previously thought^33^. Males may therefore experience selection to discriminate kin and avoid inbreeding by preferring unrelated, perhaps unfamiliar females as mates. Such information-gaining in communication networks might take place beyond the level of a signal-receiver dyad. ‘Eavesdropping’ receivers can have important implications as revealed by empirical studies on male-male assessment and mate choice in birds^34^,^35^,^36^. Likewise, male elephants might perceive information about nearby mates and adjust their behavior in response to female social vocalizations (i.e. the commonly used contact rumbles)^37^.

Here, we tested whether free-ranging male African elephants extract social information from vocalizations. We conducted playback experiments investigating the behavioral response of male subjects towards contact rumbles from familiar (from the same population) versus unfamiliar females. We examined discrimination by specific responses indicating attentive behavior, represented by the variables *ears lifted, head lifted* and *stop feeding/drinking*. In addition, we simultaneously investigated whether males show preference for the rumbles of familiar or unfamiliar females by analyzing their orientating responses including the behavioral variables *turn to speaker, face speaker* and *approach speaker* (Table 1 and Fig. 1, Supplementary Videos S1 and S2).

## Results

The elephants clearly responded to all 54 successful playback trials with attentive behavior (elephants *lifted* their *ears* and *stopped feeding/drinking*). Linear mixed model (LMM) analysis revealed that males responded for longer to the rumbles of unfamiliar females including *ear lifting (F*_(1,25)_ = 33.47, *P* = 0.000) and *stop feeding/drinking (F*_(1,25)_ = 16.766, *P* = 0.000) (Fig. 2, Table 2). The LMM revealed no significant effect for order of presentation of the stimuli (familiar – unfamiliar and reverse), i.e. no habituation or enhancement effects occurred when playing back the second trial after at least 10 minutes (*ear lifting*: *F*_(1,25)_ = 0.322, *P* = 0.575, *stop feeding*: *F*_(1,25)_ = 0.174, *P* = 0.894). Further, we tested whether rumble duration affected behavioral responses (since call duration of the two rumbles was not exactly equal). The LMM showed no significant effect on rumble duration (*ear lifting*: *F*_(1,25)_ = 0.068, *P* = 0.796, *stop feeding*: *F*_(1,25)_ = 0.001, *P* = 0.989).

The elephants spent significantly more time *lifting* their *head, facing* and *approaching the speaker* in response to the rumbles of unfamiliar females (Wilcoxon Signed Rank Tests; *P* < 0.01, Fig. 2, Table 2). No significant difference was documented in the variable *trunk lift*, which occurred infrequently in response to both stimuli types, or in the variable *turn to*. Only two male elephants responded vocally (each once) in response to a rumble of a familiar female.

We further evaluated how males responded to rumbles from the unfamiliar females of the Vienna zoo compared to those recorded from the captive (unfamiliar) South African females. The responses didn’t differ for any of the variables: *ears lifted* (multi-factorial ANOVA: *F*_6,20_ = 0.655, *P* = 0.343) and *stop feeding/drinking (F*_6,20_ = 0.639, *P* = 0.320), *head lifted* (Kruskal Wallis Test *X*^*2*^_*(6)*_ = 4.011, *P *=* *0.135), *face speaker (X*^*2*^_*(6)*_ = 5.575, *P *=* *0.095), *approach speaker (X*^*2*^_*(6)*_ = 6.851, *P *=* *0.087), *turn to speaker (X*^*2*^_*(6)*_ = 6.763, *P* = 0.091) and *trunk high (X*^*2*^_*(6)*_ = 4.426, *P *=* *0.124).

## Discussion

Our results show that male elephants can discriminate rumbles from familiar versus unfamiliar females. The males generally displayed longer attentive reactions in response to the rumbles of unfamiliar females. These behaviors, and in particular when chewing, breaking branches and similar feeding activities (sucking and swallowing in the case of drinking) are interrupted and the elephant basically pauses movement, are suggested to indicate an intensive period of listening^38^. Increased attentive behavior can be driven by importance or novelty. Animals are expected to respond longer towards stimuli that are more relevant for survival or reproductive success, for example vocalizations of their kin or social affiliates compared to unrelated animals, although which matter more can depend on the context (i.e. social versus reproductive)^39^. Alternately, they might attend longer to stimuli from unfamiliar, than to stimuli from familiar individuals, and their response could be interpreted as surprise^39^. In either case, discrimination is apparent. Our study provides clear evidence that male elephants perceive and discriminate the social information contained in long-distance female vocalizations.

In two of our playback trials, the males vocalized in response to rumbles from familiar females. The missing information about the natal families of our focal males complicates the interpretation of these interesting observations. In these two cases the familiar rumbles may have, by chance, stemmed from a female of the males’ natal family group, thus representing a vocalization of a close relative or associate that evoked a vocal response. Whether the males generally recognized individual females (in those reactions involving familiar rumbles) remains to be investigated. Acoustic cues advertising family, bond group and population identity could have allowed subjects to perform the perceptual discrimination. Males might have learned to identify vocal characteristics of the different families of their population during youth, when they were still part of the matrilineal fission-fusion system. Alternately, subjects could merely have identified the female rumbles as having been encountered before.

Beyond the attentive behaviors, males displayed significantly longer orientating responses (longer periods of *facing the speaker* and *approaching the speaker*) towards rumbles of unfamiliar females. We cautiously interpret this as increased preference. Only the variable *turn to*, an orientating response less intensive compared to *facing* or *approaching the speaker*, revealed no significant difference. In female elephants tested by McComb^20^, approaches to the loudspeaker were exclusively associated with playbacks of family or bond group members (i.e. highly familiar individuals). Their playbacks of rumbles from low association index families generally increased group cohesion and avoidance behavior^20^. These sex-dependent observations in otherwise quite similar experiments (testing discrimination of familiar and unfamiliar vocalizations) suggest that males might utilize vocal cues in mate assessment. In mammals, orientating and approach responses are often used to measure preference behavior^40^ and mate choice^41^,^42^. Orientating responses play an integral role in preference formation. Research suggests that animals, when deciding between options, typically end up choosing the items they preferentially orient toward^40^. This gaze can occur while the stimulus is present or after it has been removed, the latter causing the gaze to be fixated in the direction in which the stimulus had been present. Interestingly, gaze bias ceases following a decision, suggesting that gaze bias is the cause of preference and not its effect^40^. Though we cannot exclude that the observed orientating responses were driven by novelty, the fact that females in similar experiments preferred the stimuli of more familiar females^20^ (indicating that elephants do not automatically orientate towards novel stimuli), point to a preference rather than a novelty effect. Future research will compare the reactions of male subjects to unfamiliar female and male vocalizations in more detail in order to eliminate potential novelty effects. To examine the possibility that males use vocal signals in mate assessment, experiments similar to the one reported in this study with vocalizations that stem from known estrous females using males in musth^43^ as subjects are absolutely essential, though highly challenging. Indeed, it has been shown that the call of an estrous female is highly attractive to males in musth^28^. In addition, such experiments need to include long-term data on relatedness, spatial distribution, ranging pattern and social affiliations of focal males, which is only possible in intensively monitored populations.

In conclusion, our results provide strong evidence that male African elephants extract social information from female social rumbles. These results should help focus attention on the cognitive abilities of male elephants, including vocal perception and discrimination. Future research needs to examine the fitness consequences for male elephants of developing knowledge and, maybe, long-term recognition of signals from a dispersed population of potential mating partners in a highly competitive social and reproductive environment.

## Methods

### Study site and subjects

The study was conducted at the Addo Elephant National Park (AENP) Eastern Cap Province, South Africa between June and September 2015 and 2016. The enclosed elephant habitat of the AENP is about 380 km^2^ with no access to other elephant populations via migration corridors. It is situated in a succulent thicket vegetation type^44^ that is evergreen and nutritious^45^. In 2008, the elephant population numbered 481 individuals with an annual rate of increase of 5.81%^46^,^47^. Accordingly, an estimated 700 elephants lived in the park in 2015. The female population comprises eight family groups, the A, B1 and B2, H, L, M, P and the R family^46^. Information about family affiliation is known for the female AENP population but missing for most adult males. Four adult males were introduced into the AENP in 2002 and 2003 from Kruger National Park; otherwise the population is indigenous. The total number of independent (above 14 years) and mature bulls (above 25 years) living in Addo is not known, but we have so far individually identified 55 mature males.

The subjects for playback experiments were 27 adult medium (25–35 years) and large (>35 years) males^11^, individually identified based on notches and holes on both of their ears. Age was appraised based on a combination of visual cues such as overall size and appearance, the tusk girth and head shape^48^. For 5 males, the approximate age was known.

### Ethic statement

The experimental protocols were approved by the Animal Welfare Board of the Faculty of Life Sciences, University of Vienna, and the research committee of the South African National Parks (SanParks). This study complies with all applicable Austrian and South African laws and was conducted in accordance with the Guidelines for the Treatment of Animals in Behavioral Research and Teaching^49^.

### Recording equipment

For acoustic recordings of playback stimuli and during playback experiments we used an omni-directional Neumann microphone (KM 183) modified for recording frequencies below 20 Hz (flat recording down to 5 Hz) connected to a 722 Sound Device HDD recorder at a 48 kHz sampling rate and 16-bit amplitude resolution.

### Playback equipment

**Figure 1:**
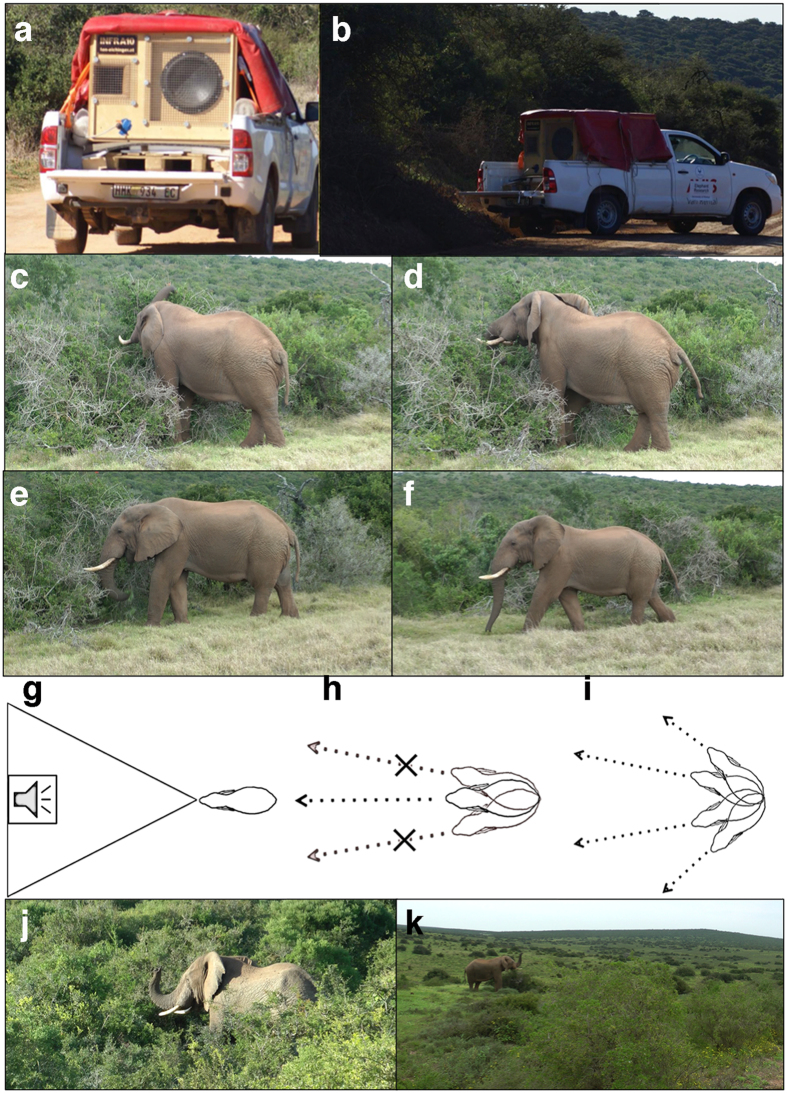
Photographs and illustrations of playback equipment in the field and behavioral responses used to analyze reactions of the male elephants to the playback stimuli. (**a**) Speaker on vehicle, (**b**) speaker conducting playback, (**c**) relaxed elephant feeding prior to playback (**d**) *ears lifted and head lifted* in reaction to playback, (**e**) position of relaxed head before playback (head is below shoulder level), (**f**) *head lifted, ears lifted* while *approaching the speaker* in response to a playback, (**g**) *approach speaker*, (**h**) *face speaker*, (**i**) *turn to speaker*, (**j**, **k**) *trunk high*.

**Figure 2:**
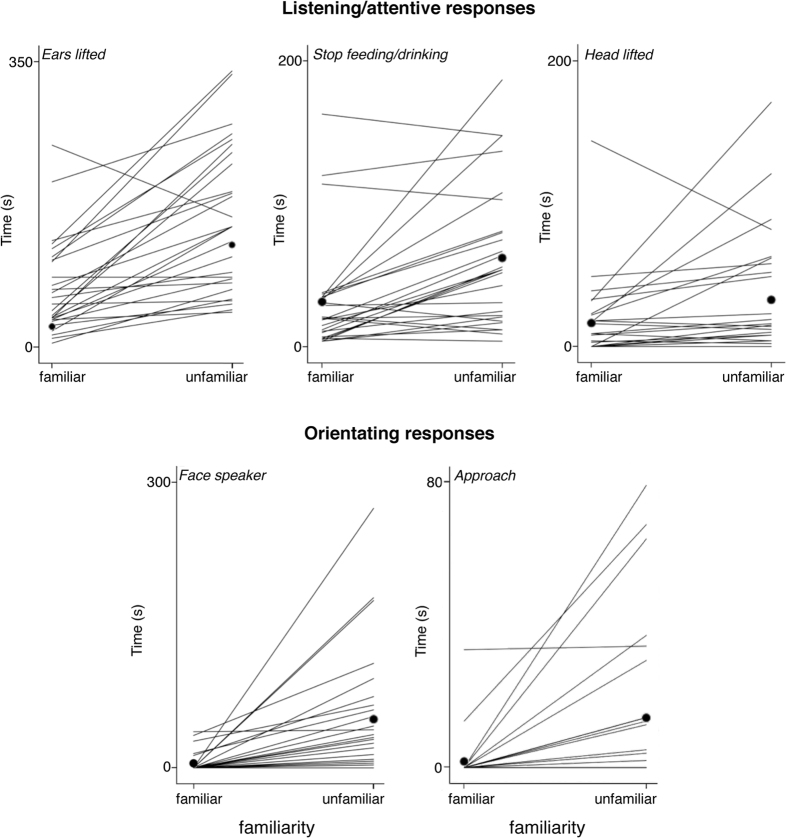
Responses (in seconds) of each male elephant (and the means indicated by black dots) to playbacks of familiar and unfamiliar female rumbles, including listening/attentive and orientating responses.

We used our custom-built subwoofer INFRA10 and a JL Audio HD1200/1 audio amplifier for playback experiments linked to a 722 Sound Device HDD recorder (see Supplemental information for details). This subwoofer is designed for playback frequencies giving a flat response from 10–200 Hz at peak sound pressure levels (SPL) measured at 1 m from the source of 110 dB at 10 Hz (referenced to 20 μPa). The dimensions of the speaker are 198 × 166 × 171 mm. The speaker is battery supplied and transported on an off-road vehicle (Fig. 1a,b).

### Playback stimuli

For playback stimuli (a stimulus = one rumble) we used rumbles of adult female elephants. Adult females were defined having reached 11 years of age, the mean age of first conception^9^,^20^. The familiar rumbles (*N* = 18) came from 18 individually identified females of 7 family units (2–3 individuals per family) recorded in the AENP in 2011 and 2012. The rumbles of unfamiliar females (*N* = 27) were recorded from seven individuals at Pilanesberg Back Safaris, Adventures with Elephants (Bela Bela), Elephant Whisperers (Hazyview) in 2014, all South Africa, and at Vienna Zoo, Austria, in 2014 and 2015 (those two females originate from South Africa and Zimbabwe) (see Supplemental Tables S1 and S2 for detailed information on the rumbles used for the experiments).

All rumbles were recorded in calm social contexts during browsing, where individuals divide up and decentralize. When engaged in feeding and browsing, female elephants will occasionally rumble to maintain vocal contact with other group members (also in the zoo), and solely these rumbles were selected. In order to correctly assign vocalizations to individuals, we focused on particular elephants for a certain time period and recorded in close distance (<30 m). We also used video recordings to verify vocalizing individuals during data annotation.

**Figure 3:**
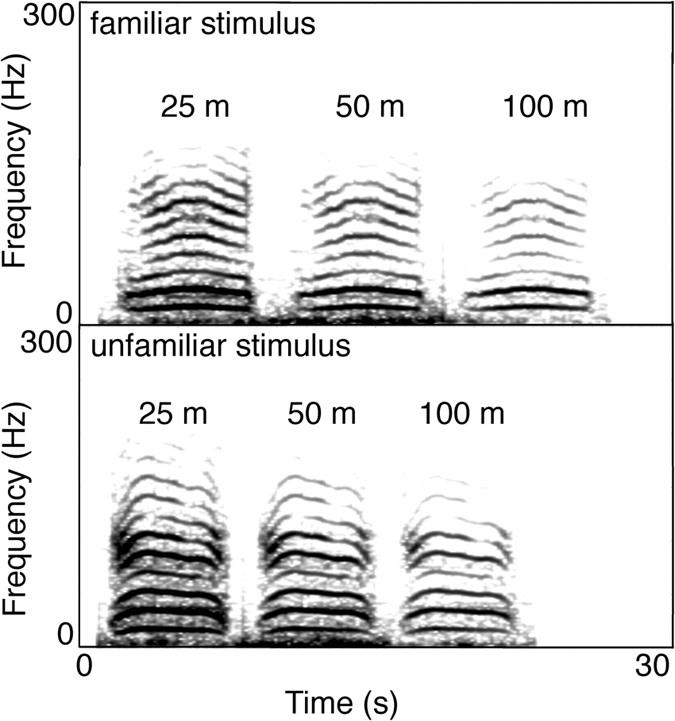
Spectrograms of propagation experiments of two female social rumbles at 25, 50 and 100 m, respectively. The upper harmonics persist well over 100 m. Based on these experiments, we determined that the playback stimuli could be displayed between 40 and 100 m without relevant information loss.

We selected high quality, nasal rumbles^50^ with a clear fundamental frequency and upper harmonics including formant 1 and 2 recorded in less than 30 m distance. We filtered any sound below 8 Hz and above 300 Hz to diminish unnecessary background noise in PRAAT^51^ (using a 10 Hz smoothing). We evaluated previously that the selected rumbles had no sound energy above 300 Hz. All sound samples were normalized to 99% peak amplitude with Audacity (version 2.01; Effect Normalize amplitude to −1 dB) and played back at 103 dB ± 1 dB rel 1 meter (measured with an NTI – AL1 sound pressure level meter equipped with an NTI MiniSPL microphone). In order to verify the quality of the playback rumbles, we conducted propagation experiments outside the hearing range of the elephants. We played back rumbles from familiar and unfamiliar females (*N*_calls_ = 10) at distances of 25, 50 and 100 meter (at the distances at which the subjects were supposed to receive them) to control fidelity of sound reproduction (Fig. 3, see Supplementary information online for measurements of environmental conditions). Since social information ought to be consistently transmitted via the playbacks, we aimed at a playback distance where upper harmonics of the rumble until at least the second formant were clearly visible in the spectrogram (this, at a minimum, requires pronounced harmonics up to 150 Hz). Based on these experiments, we determined that the playback distance should be ideally between 40 and 100 m.

### Experimental set-up

The team used three vehicles, one with the INFRA10 subwoofer, one observer car (recording behavioral reactions) and one tracking car (searching for males meeting playback criteria) with radio contact and mobile phones. Male subjects needed to be solitary (no other male within visible range, and no female group within 1 km). We started playback trials only if a bull was browsing calmly or drinking at a waterhole, in each case facing the opposite direction of the speaker. This enabled reactions such as *turn to speaker, face speaker*, or *approach speaker* to be best identified. The speaker was positioned in line to the elephant at distances ranging from 40–100 m, always hidden behind bushes (so that the elephant could not see the speaker, even if turning towards it). The observer car was positioned such that it could record the behavior of the elephant during playbacks. We abandoned five trials because of disturbances due the presence of another vehicle, and three trials because another male appeared.

Each male was exposed to two consecutive playback trials. Per trial we played back one stimulus (=one rumble), either a rumble of a familiar, or an unfamiliar female, counterbalancing the order across subjects. The two trials were separated by a minimum of 10 (range = 10–22, median = 12) minutes, beginning when the elephant stopped reacting (see Table 1 for definition and Supplementary Video S3). The rumbles for each trial were randomly selected (meaning that we did not select for specific individuals to be compared) from the available recordings (Supplementary Table S1). The only aspect we adjusted for was rumble duration, in order to playback rumbles of approximate similar duration in the two consecutive trials. The mean duration ± SD of the familiar rumbles was 4.29 ± 1.147 s (minimum = 2.7 s, max = 6.8 s), the mean duration ± SD of unfamiliar rumbles was 4.27 ± 1.03 s (minimum = 2.6 s, maximum = 6.4 s). Supplementary Table S2 gives the ID and the duration of the rumbles used as stimuli for each playback trial. Each rumbles was used only once, except for 9 familiar ones that had to be used twice with a minimum of 16 days in between. We successfully conducted two playback trials (testing one rumble of a familiar and one of an unfamiliar female) on 27 males.

### Data analyses

Videos were analyzed frame-by-frame (frame = 20 ms) using Solomon Coder Software Version beta 15.11.19^52^. We measured listening/attentive responses and orientating responses (Table 1).

Attentive responses include *ears lifted, head lifted* and *stop feeding/drinking*. An elephant rarely stands still except when attentive or listening. Poole & Granli^53^ write that usually some parts of the body, ears, trunk or tail are in motion, and the head is in a relaxed position below the level of the shoulders. When listening, an elephant often stands still, the body and extremities cease moving as it simultaneously raises its head and stiffens its ears.

AS analyzed the videos and a random subset (15 videos = 19.4%) of the trials were double coded by an independent observer, providing an inter-observer reliability of *r*_*s*_ = 0.934 for *ears lifted, r*_*s*_ = 0.992 for *stop feeding/drinking, r*_*s*_ = 0.894 for *head lifted, r*_*s*_ = 0.975 for *face speaker*, and *r*_*s*_ = 0.943 for *approach speaker* (all *P* ≤ 0.001) measured by Spearman’s rho correlation.

### Statistical analysis

In order to test for the effect of familiarity on the attentive variables *ears lifted* and *stop feeding/drinking*, we ran two LMMs with familiarity as a fixed factor, subject and stimuli (rumble) ID as random factors (using Restricted Maximum Likelihood Estimation). The models also tested for a fixed effect of rumble presentation order (familiar or unfamiliar first) and rumble duration, to test whether the elephants, in general, responded more intensive towards the longer rumble (for this, the two rumbles played back per subject were categorized as being ‘the longer’ or ‘the shorter rumble). Although these did not attain significance for either variable, they remained in the model because removing them did not improve model quality (using Akaike’s Information Criterion). Due to parametric assumption violations (normality of residuals verified using Kolmogorov-Smirnov and Shapiro-Wilk tests), we performed nonparametric Wilcoxon signed-rank tests on the variables *head lifted, approach speaker, face speaker, turn to speaker* and *trunk high* to test discrimination of the rumbles of familiar versus unfamiliar females. In order to test whether these variables correlated with rumble presentation or rumble duration (e.g. potential habituation or a generally increased arousal in response to the second, or the longer stimulus), we conducted Spearman rank correlation tests.

In addition we evaluated whether male responses to the rumbles from the Vienna zoo elephants differed compared to the rumbles recorded from the captive South African females. We conducted an independent multi-factorial ANOVA (since each male was tested with only one unfamiliar stimulus, we had a between-group design) on the variable *ears lifted and stop feeding/drinking*. We designated these as dependent variables, identity of the unfamiliar female as fixed factor, the identity of the focal elephant as random factor. We used Kruskal Wallis Test to compare the variables *head lifted, approach speaker, face speaker, trunk high* and *turn to speaker*.

All tests were two-tailed, alpha was set at 0.05 and Bonferroni-corrected.

## Additional Information

**How to cite this article:** Stoeger, A. S. and Baotic, A. Male African elephants discriminate and prefer vocalizations of unfamiliar females. *Sci. Rep.* **7**, 46414; doi: 10.1038/srep46414 (2017).

**Publisher's note:** Springer Nature remains neutral with regard to jurisdictional claims in published maps and institutional affiliations.

## Supplementary Material

Supplementary Information

Supplementary Video 1

Supplementary Video 2

Supplementary Video 3

## Figures and Tables

**Table 1:** The description and sampling method of the behaviors used to analyze subjects’ response to playback stimuli.

Category	Measurable behavior*	Description	Sampling method
Listening/attentive	*Ears lifted*	The ears are lifted and slightly extended at an angle of about 45°. We stopped measuring once the elephant relaxed its ears on the shoulders and remained in this position (not lifting the ears again) for 5 seconds (Supplemental video 3).	Measured in seconds (s); Continuous
	*Head lifted*	Lifting the head above shoulder level (Supplemental Figure 3). We stopped measuring “head lift” if the head was back in line with, or below the shoulders.	Measured in seconds (s); Continuous
	*Stop feeding/drinking*	The elephant ceases all feeding or drinking activities, stops chewing or swallowing, breaking branches or picking grass, sucking in water; Supplemental video 1 and 2). We stopped measuring this variable as soon as the elephant resumed one of the mentioned activities).	Measured in seconds (s); Continuous
Orientating response	*Approach speaker*	The elephant approaches the speaker at a ≈40° angle. We measured approach once the elephant moved at least three steps forward. Thus, for example, two steps in the direction of the speaker (perhaps to reposition himself) were not considered as approach.	Measured in seconds (s); Continuous
	*Face speaker*	The elephant positions himself to face into the direct line of the speaker.	Measured in seconds (s); Continuous
	*Turn to speaker*	The elephant orientates himself into the direction of the sound source, but not directly facing the speaker (Supplemental video 1 and 2)	Frequency of occurrences
Other behaviors	*Trunk high*	The trunk tip is lifted above the level of the tusks. The elephant does not have to be focused into the direct line of the speaker.	Measured in seconds (s); Continuous
	Vocal response**	Any vocalization in response to our stimulus (that occurred within 5 min following the playback).	Frequency of occurrences
End of reaction*		Following an initial reaction, the elephants first resumed feeding/drinking and lowered the head. *Ears lifted* was the behavior observed longest. When the ears relaxed (ears touching the shoulder), and remained relaxed for 5 seconds, we stopped measuring the behavior (=end of reaction).	

^*^See online supplemental information for video examples.

^**^Not entered into the analysis because of sporadic, irregular occurrence.

**Table 2:** Statistics from playbacks on 27 individuals on the behavioral responses to social rumbles from familiar versus unfamiliar females.

Variables	Test on familiarity							Test on stimuli order		Test on stimuli duration	
	Mean (s) ± s.e.m	Linear Mixed Model						Linear Mixed Model			
	*fam*	*unfam*	*Estimate*	*SE*	*df*	*F*	*P*	*F*_*(1,25)*_	*P*	*F*_*(1,25)*_	*P*
*Ears lifted*	71.8 ± 11.2	157.7 ± 17.2	−85.889	14.32	1,25	33.471	0.000	0.322	0.575	0.068	0.796
*Stop feeding/drinking*	31.56 ± 7.5	62.3 ± 9.4	−30.741	7.52	1,25	16.766	0.000	0.174	0.894	0.001	0.989
			**Two-tailed Wilcoxon Signed Rank Tests**					**Spearman’s rho**			
			***Z***		***P***			***r***_***s***_	***P***	***r***_***s***_	***P***
*Head lifted*	16.4 ± 5.5	32.7 ± 8.3	−2.957		0.003			−0.053	0.663	0.050	0.722
*Approach speaker*	1.7 ± 1.2	13.9 ± 4.5	−3.181		0.002			0.065	0.590	−0.101	0.467
*Face speaker*	4.7 ± 2.1	51.1 ± 12.7	−4.107		0.001			0.097	0.423	−0.130	0.350
*Trunk high*	0.8 ± 0.6	2.4 ± 1.2	−1.322		0.186			0.022	0.091	−0.106	0.447
	**Frequency of occurrence**										
*Turn to speaker*	16	20	−1.609		0.108			−0.022	0.857	−0.011	0.936
