# Corrigendum: Male African elephants discriminate and prefer vocalizations of unfamiliar females

**DOI:** 10.1038/srep46892

**Published:** 2017-08-24

**Authors:** Angela S. Stoeger, Anton Baotic

Scientific Reports 7: Article number: 46414; 10.1038/srep46414 published online: 04 19 2017; updated: 08 24 2017.

This Article contains an error in the Acknowledgements section, where

“This research was funded by the FWF, the Austrian Science Fund [**P26488-N30**].”

should read:

“This research was funded by the FWF, the Austrian Science Fund [**P26448-N30**].”

