# Supplementary material for: Male African elephants discriminate and prefer vocalizations of unfamiliar females
Source: Sci Rep. 2017 Apr 19;7:46414. doi: 10.1038/srep46414 (PMC5395942; doi:10.1038/srep46414)
Supplement: Supplementary Information [file srep46414-s1.pdf]

Supplemental Information

Male African elephants discriminate and prefer vocalizations of unfamiliar females

Angela S. Stoeger[a]* & Anton Baotic[a]
[a] Mammal Communication Lab, Department of Cognitive Biology, Althanstrasse 14, 1090, University of Vienna, Austria

*corresponding author: angela.stoeger-horwath@univie.ac.at
tel.: ++43 1 427776101
fax.: ++43 1 42779761

1) **Additional Information on playback equipment**
2) **Environmental conditions during propagation experiments**
3) **Supplemental Table S1 giving the total number of stimuli used throughout the study**
4) **Supplemental Table S2 giving the ID and the duration of each stimulus used for playback.**
5) **Information on supplemental videos**

1) Playback equipment: additional specifications

The playback equipment was custom built. Technical specifications:
Nominal impedance: 4 Ohm
Frequency range: 10 Hz to 200 Hz
200 Hz to ca. 3000 Hz with reduced accuracy and increased beaming on axis
Maximum sound pressure level: 110 db SPL from 10 Hz to 200 Hz at the recommended maximum input power of 400 W
Recommended maximum power: 400 W (40 V)
Absolute maximum power, not recommended for operation: 2000 W (89 V)
Outer dimensions – 1180 mm width x 1730 mm length x 980 mm height
Weight approximately 300 kg

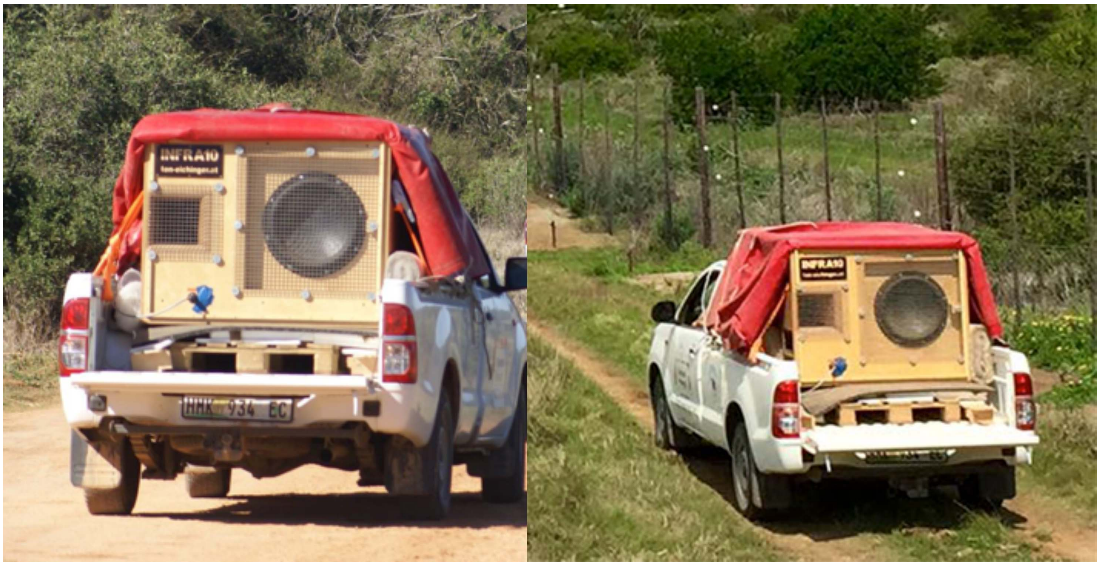

Supplemental Figure S1. Photographs of our subwoofer on the vehicle at the Addo Elephant National Park. In order to minimize noises due to low-frequency vibrations during playback, we use isolating, sound-proofing material (such as anti-vibration rubber mats).

2) Propagation experiments

In order to verify the quality of the playback stimuli, we conducted propagation experiments outside the range of hearing of the elephants. We played back 4 familiar and 6 unfamiliar stimuli (N_{calls} = 10) at distances of 25, 50 and 100 m to control fidelity of sound reproduction.

Environmental conditions during propagation experiments:

At 25 m distance:

Temp °C	18.0
Humidity %	55
Speed m/s	< 1.2

At 50 m distance:

Temp °C	20.4
Humidity %	55.5
Speed m/s	< 0.1

At 100 m distance

Temp °C	24.5
Humidity %	41.9
Speed m/s	< 0.4

3) Supplementary Table S1. Total number of stimuli (a stimulus = one rumble) used throughout the study (indicating the number of individuals and rumbles per individuals available). Each male was exposed to two consecutive playback trials. Per trail we played back a rumble of a familiar, or an unfamiliar female, counterbalancing the order across individuals. For example, we started with a rumble from Mussina (a unfamiliar female), followed by a rumble from a familiar individual of the B1 family. Stimuli were used only once, except for 9 familiar stimuli that had to be used twice with a minimum of 16 days in between.

**Stimuli pool for playback trials**	
**Familiar (AENP) *N*=18**	**Unfamiliar (*N* =27)**
B1 family ($N_{ind=calls}$=3)	Chickwenya, age 29 (N_{calls} = 5)<br>Pilanesberg
B2 family ($N_{ind=calls}$=3)	Mussina, 12 (N_{calls} = 4)<br>Bela Bela
H family ($N_{ind=calls}$=3)	Nuanedi, age 13 (N_{calls} = 3)<br>Bela Bela
L family ($N_{ind=calls}$=2)	Shan, age 16, (N_{calls} = 4)<br>Bela Bela
M family ($N_{ind=calls}$=2)	Andile, age 14, (N_{calls} = 4)<br>Hazyview
P family ($N_{ind=calls}$=3)	Tonga, 31 (N_{calls} = 3)<br>Vienna Zoo (originally from South Africa)
R family ($N_{ind=calls}$=2)	Drumbo, 41 (N_{calls} = 4)<br>Vienna Zoo (originally from Zimbabwe)

4) Supplemental Table S2 gives the file name (the ID of the stimulus) and it's duration for each trial. As mentioned in the main paper, the stimulus represents either a rumble of a familiar (1), or a rumble of an unfamiliar (X) female. 1X means that a rumble of a familiar female was played in the first trial, followed by a rumble of an unfamiliar female in the second trial (X1 thus means unfamiliar rumble first, familiar rumble second).
Explanation for familiar stimuli: Stimulus ID: B1, B2, L, H, P, M and R represent rumbles recorded of individuals belonging to the corresponding family units of the Addo Elephant National Park. E.g. B1_1_a0584_mod.wav indicates a rumble from the individual 1 of the B1 group. The numbers with a letter in front e.g. - a0584- indicates the original ID code of the recording, and 'mod' means that the file has been modified for playback (as described in the main paper).
Explanation for unfamiliar stimuli: pi: recordings of the female (Chickwenya) located at Pilanesberg NP, ef: recordings of females at Bela Bela (based on the ID number we know the individual), ew: recordings of Andile at Hazyview, W: recordings of the females at the Vienna Zoo (T = Tonga, D = Drumbo).

**Male subjects**	**1. Trial**		**2. Trial**	
	**Stimulus ID**	**Duration**	**Stimulus ID**	**Duration**
**Subject 1_1X**	B1_1_a0584_mod.wav	6.1 s	ef109_mod.wav	5.9 s
**Subject 2_X1**	pi222_mod.wav	5.0 s	P_2_a0176_mod.wav	4.2 s
**Subject 3_1X**	L_1_a0699_mod.wav	3.6 s	ew120_mod.wav	3.9 s
**Subject 4_X1**	pi256_mod.wav	2.6 s	H_3_a0387_mod.wav	2.7 s
**Subject5_1X**	H_1_a0385_mod.wav	3.5 s	pi040_mod.wav	3.8 s
**Subject 6_X1**	W1_D_a0776_mod.wav	4.1 s	R_2_a0681_mod.wav	4.3 s
**Subject 7_1X**	P_1_a0186_mod.wav	4.1 s	ef015_mod.wav	3.6 s
**Subject 8_1X**	B1_2_a0582_mod.wav	6.8 s	W_D_w0765_mod.wav	6.4 s
**Subject 9_X1**	R_1_a0686_mod.wav	3.1 s	ef458_mod.wav	3.0 s
**Subject10_1X**	L_2_a0712_mod.wav	6.2 s	W_T_w0211_mod.wav	6.0 s
**Subject 11_X1**	ef133_mod.wav	4.5 s	H_2_a0411_mod.wav	4.8 s
**Subject 12_1X**	B2_1_a0014_mod.wav	4.3 s	ef134_mod.wav	3.6 s
**Subject 13_X1**	W_T_w0801_mod.wav	4.8 s	P_2_a0176_mod.wav	4.2 s
**Subject 14_1X**	H_3_a0387_mod.wav	2.7 s	pi254_mod.wav	3.3 s
**Subject 15_1X**	B2_2_a0018_mod.wav	4.3 s	ef135_mod.wav	4.0 s
**Subject 16_X1**	ef293_mod.wav	2.7 s	M_1_a0104_mod.wav	2.8 s
**Subject 17_1X**	B1_3_a0437_mod.wav	5.4 s	pi249_mod.wav	5.0 s
**Subject 18_1X**	P_3_a0211_mod.wav	3.2 s	W_T_w0340_mod.wav	3.7 s
**Subject 19_X1**	W_D_w0334_mod.wav	3.4 s	B2_3_a0543_mod.wav	3.5 s
**Subject 20_1X**	B1_1_a0584_mod.wav	6.1 s	ef243_mod.wav	5.9 s
**Subject 21_X1**	W_D_w0512_mod.wav	3.6 s	M_2_a0090_mod.wav	3.5 s
**Subject 22_1X**	P_1_a0186_mod.wav	4.4 s	ef108_mod.wav	3.8 s
**Subject 23_X1**	ew350_mod.wav	5.2 s	L_2_a0712_mod.wav	4.8 s
**Subject 24_1X**	H_2_a0411_mod.wav	4.8 s	ef218_mod.wav	4.9 s
**Subject 25_X1**	ew300_mod.wav	4.2 s	B2_1_a0014_mod.wav	4.3 s
**Subject 26_1X**	M_1_a0104_mod.wav	2.9 s	ew156_mod_.wav	3.3 s
**Subject 27_X1**	ef456_mod.wav	5.0 s	B1_3_a0437_1_mod.wav	5.4 s

5) Supplementary videos

Supplemental Video 1: Shows reactions in response to an unfamiliar stimulus (*ears lifted*, *head lifted*, *stop feeding*, *turn to speaker*, *trunk high*, and *face speaker*).

Supplemental Video 2: Shows reactions in response to another unfamiliar stimulus (*ears lifted*, *head lifted*, *stop feeding*, *turn to speaker*, *approach speaker*).

Supplemental Video 3: Gives examples for ear relaxation (end of *ears lifted*). Usually, following an initial reaction, the elephants first resumed feeding/drinking and lowered the head. *Ears lifted* was the behavior observed longest. When the ears relaxed (ears touching the shoulder), and remained relaxed for 5 seconds, we stopped measuring the behavior (=end of reaction).
